# Evaluation of the Expression of Virulence Factors of *V. cholerae* After Interaction With the Human Colon Adenocarcinoma (Caco-2) Cell Line

**DOI:** 10.1155/cjid/9936375

**Published:** 2025-07-22

**Authors:** Mohammadreza Kheradmand, Masoumeh Saberpour, Bita Bakhshi, Mahboube Bahroudi

**Affiliations:** ^1^Department of Bacteriology, Faculty of Medical Sciences, Tarbiat Modares University, Tehran, Iran; ^2^Department of Influenza and Other Respiratory Viruses, Pasteur Institute of Iran, Tehran, Iran

**Keywords:** BHI broth, Caco-2 cells, *V*. *cholerae*, virulence factors

## Abstract

Cholera remains a global challenge, and understanding how *V*. *cholerae* adapts to environmental condition is essential for innovating new management strategies. This research aims to examine the expression of *ctxAB*, *tcpA*, and *hlyA* genes in *V*. *cholerae* (El Tor and classical biotypes) after interaction with Caco-2 cells compared to brain heart infusion (BHI) broth. After assaying of viability of Caco-2 cells against *V*. *cholerae* at multiplicity of infection (MOI) of 10, 20, 50, and 80, the number of bacteria attached to Caco-2 cells was determined using the adhesion assay. To conduct a valid comparison, an equivalent number of bacteria that attached to Caco-2 cells was inoculated into BHI broth. The expression of *ctxAB*, *tcpA*, and *hlyA* genes in *V*. *cholerae* (El Tor and classical biotypes) was assayed using the real-time PCR method. After interaction of Caco-2 cells with *V*. *cholerae*, the expression of the genes *ctxAB*, *tcpA*, and *hlyA* in the El Tor biotype increased by 2-, 1.02-, and 7-fold, respectively, while in the classical biotype, it increased by 6-, 2-, and 13-fold, respectively. The expression of the genes *ctxAB* and *hlyA* was significantly increased in Caco-2 cells in both biotypes. There was a significant increase in the expression of the gene *tcpA* in the classical biotype, while no significant increase was detected in the expression of this gene in the El Tor biotype. Caco-2 cells induced the highest increase in *hlyA* gene expression in the classical biotype, while there was no significant effect on *tcpA* gene expression in the El Tor biotype of *V*. *cholerae*. In conclusion, *V*. *cholerae* showed greater pathogenicity in the Caco-2 cells. Knowing the behavior of *V*. *cholerae* in different conditions can lead to create innovative strategies for combating and managing cholera.

## 1. Introduction

Cholera is a major health challenge with high mortality rates worldwide, especially in developing nation [1]. Cholera is a disease characterized by severe watery diarrhea caused by *Vibrio* (*V*). *cholerae*. It exists in contaminated water and displays diverse pathogenic behaviors under various environmental conditions [2]. After exposure of *V*. *cholerae* to unfavorable environments, it modifies its pathogenic behavior by regulating the expression of virulence factors, thereby adapting to adverse conditions and demonstrating increased resistance [3]. So, analyzing of the behavior of this pathogen in different environments and monitoring the expression of its virulence factors help us develop effective strategies for managing this organism. *V*. *cholerae* is a gram-negative bacterium with many virulence factors including cholera toxin (CT) (*ctxAB*), toxin-coregulated pilus (*tcpA*), hemolysin A (*hlyA*), zonula occludens toxin (Zot), accessory cholera enterotoxin (Ace), flagella, outer membrane protein U (OmpU), haemagglutinin/protease (HapA), and ToxR-activated gene- A (TagA). The main virulence factors of this bacterium are CT (*ctxAB*) that produced by a lysogenic phage (CTXΦ), and it alters signaling transduction pathways in the host and leads to diarrhea, toxin-regulated pilus (*tcpA*) that is a major adhesion factor for colonization of the intestinal epithelium, and hemolysin-encoded by the *hlyA* gene [4]. These factors are contributed to the high resistance of bacteria in diverse environmental conditions, including aqueous and nonaqueous environments, and adverse environmental conditions such as nutrient deficiencies, alterations in acidity, and fluctuations in temperature [5]. *V*. *cholerae* regulates its gene expression in response to adverse conditions. This adaptive process enhances effective colonization, survival, and virulence [3]. Considering the important role of key virulence factors in the persistence of *V*. *cholerae* across different environments, assessing the interaction of *V*. *cholerae* with various environments is essential. In this study, two different environments including Caco-2 cells and brain heart infusion (BHI) broth medium were used. Caco-2 cells derived from human colon adenocarcinomas have the ability to spontaneously differentiate and form a monolayer in cell culture. After achieving maximum differentiation, these cells display the morphological and functional characteristics of enterocytes, so Caco-2 cells serve as an ideal model system for further evaluation of the pathogenesis of *V*. *cholerae* [6]. Moreover, BHI broth medium provides an ideal environment for *V*. *cholerae* due to its rich nutrient composition. Few studies exist in this field. For example, interactions of Caco-2 cells with *Clostridium perfringens* significantly increased the expression of the *Clostridium perfringens* enterotoxin (*Cpe*) gene compared to LB broth medium [7]. Moreover, some researchers focused on evaluating the impact of essential minerals, such as zinc (Zn), selenium (Se), and manganese (Mn) in the Caco-2 cell environment, on the expression of virulence factors of *V*. *cholerae* [8]. So far, no research has assessed the expression of virulence genes *V*. *cholerae* including *ctxAB*, *tcpA*, and *hlyA* following interaction with Caco-2 cells. Therefore, this study aimed to evaluate of the expression of *ctxAB*, *tcpA*, and *hlyA* genes of *V*. *cholerae* (El Tor and classical biotypes) in two conditions of Caco-2 cells (similar to the intestinal environment) and BHI broth (nutrient-rich medium) environments. It will focus on assessing how *V*. *cholerae* adapts to Caco-2 cell environments and identifying which pathogenic factors of *V*. *cholerae* are effective in adapting to this environment.

## 2. Materials and Methods

### 2.1. Growth Condition of *V*. *cholerae*

In the current study, two strains of *V*. *cholerae* (a classical biotype; ATCC 14035 and a clinical El Tor biotype) were obtained from the laboratory archive in the Microbiology Department of Tarbiat Modares University. These strains were previously confirmed using both phenotypic and molecular techniques. After growing *V*. *cholerae* on the surface of thiosulfate citrate bile salts sucrose (TCBS) agar (Merck, Germany), a single colony from each of the strain was initially cultured on BHI agar (Merck, Germany), incubated at 37°C for 24 h, and subsequently subcultured in BHI broth (Merck, Germany) at 37°C for an incubation time of 24 h. For preparing of the bacterial suspension, a volume of 300 μL from the bacterial suspension was transferred into fresh BHI broth medium and incubated at 37°C for 4 h. After 4 h, the concentration of the resulting bacterial suspension was evaluated by measuring its optical density (OD) at 625 nm. OD of the bacterial suspension was determined using the *0*.*5 McFarland standard*, equivalent to 0.1, containing 1.5 × 10^8^ colony forming unit (CFU)/mL. This number of bacteria from each of the two strains was used to inoculate Caco-2 cells [9].

### 2.2. Preparation of Caco-2 Cells

Caco-2 cells were purchased from the National Center for Genetic and Biological Resources of Iran (IBRC10094). The cells were cultured in a T75 flask (SPL, Korea) containing Dulbecco's Modified Eagle Medium (DMEM) (Gibco, USA) enriched with 10% fetal bovine serum (FBS) (Gibco, USA), 1% antibiotics (penicillin: 100 U/mL and streptomycin: 100 mg/mL) (Gibco, USA), and 2 mM L-glutamine (DNA Biotech, Iran). The cells were then incubated with 5% CO2 at 37°C for 48 h. When the cells reached 80%–90% confluency, they were subcultured into a new flask [10].

### 2.3. Evaluation of Cytotoxicity of *V*. *cholerae* on Caco-2 Cells

Persistence of Caco-2 cells was separately assessed after exposure to four multiplicities of infection (MOIs; 10, 20,50, and 80) of the *V*. *cholerae* strain (classical biotype; ATCC14035) and the *V*. *cholerae* strain (El Tor biotype) using MTT solution (3-(4,5-dimethylthiazol-2-yl)-2,5-diphenyl tetrazolium bromide) (DNA Biotech, Iran). Briefly, 10^4^ cells were seeded in each well of a 96-well culture plate (SPL, Korea), and the plates were incubated overnight at 37°C with 5% CO2. After the cells reached 80% filling, each well was washed three times with sterile phosphate-buffered saline (PBS) 1X. Subsequently, classical and El Tor biotypes of *V*. *cholerae* at MOIs 10, 20,50, and 80 were added to the cells and incubated for 2 h at 37°C with 5% CO2 [11]. In the control cells, the medium was not changed. After this time, the medium was aspirated from each well and replaced with DMEM medium containing 10% FBS. Then, the cells were incubated for 3 h at 37°C with 5% CO2. Following this time, the medium was eliminated, and 100 μL of MTT reagent was transferred to each well. Next, the cells were placed in a 37°C incubator containing 5% CO2 for 4 h in the dark. After 4 h, the MTT regent was removed from the cells. Finally, 100 μL of dimethyl sulfoxide (DMSO) (Sigma Aldrich, USA) was transferred to each well to dissolve the formazan. Then, OD of purple formazan was measured at 570 nm using an enzyme-linked immunosorbent assay (ELISA) reader (800 TS, BioTek, Winooski, Vermont, USA). The experiments were repeated three times, and the data are presented as the mean ± SD [10].

### 2.4. Evaluation Attachment of *V*. *cholerae* to Caco-2 Cells

For assessment of attachment of *V*. *cholerae* to Caco-2 cells, the 10^5^ cells seeded into a 12-well culture microplate (SPL, Korea) enriched to 10% FBS without antibiotics. After 24 h, when the cells reached to 80%–90% confluency, the medium was removed from each well, and then, the cells were washed three times with sterile PBS (1X) (Gibco, USA). Then, *V*. *cholerae* at MOI 10, which lead to the best cell persistence, subjected to each well and incubated at 37°C for 2 h in a 5% CO2 atmosphere. Following this time, each well was washed with sterile PBS (1X) to remove nonadherent bacteria to the cells. Then, 200 μL of 0.1% Triton X100 solution (Sigma-Aldrich, Germany) was added to each well and incubated at 20°C for 15 min. Following cell disruption with 0.1% Triton X-100, the bacterial suspensions were serially diluted (1:1000, 1:10,000, and 1:20,000). Next, 50 μL from each dilution was cultured on three BHI agar plates and then incubated for 24 h at 37°C. The plates were subsequently counted, and the average number of colonies was determined [12, 13].

(1) $$\begin{array}{c} \text{Attachment efficiency}=\frac{\text{The number of attached bacteria}}{\text{The number of initial inoculated bacteria}}\times 100. \end{array}$$

### 2.5. Extraction of RNA and Synthesis of Complementary DNA (cDNA)

**Figure 1:**
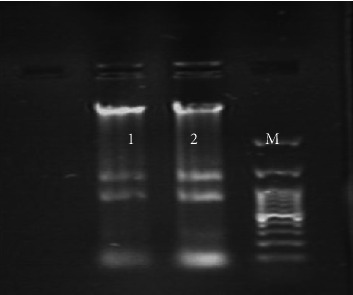
An agarose gel electrophoresis image of extracted RNA to demonstrate RNA integrity. Lanes 1 and 2: RNA extracted from *V*. *cholerae* strains, M: 100-bp molecular size marker.

RNA extraction from Caco-2 cells was performed according to the protocol RNA Miniprep Super Kit (Bio Basic, Canada). For RNA extraction from Caco-2 cells, a 12-well microplate (SPL, Korea) was prepared. Briefly, after counting cells, 3 × 10^5^ cells were seeded into each well of culture plates containing 500 μL of DMEM enriched with 10% FBS and without antibiotics. The cells were incubated with 5% CO2 at 37°C for 24 h. Following this time, when the cells reached 80%–90% confluency, the medium was aspirated, and the cells were washed three times with sterile PBS (1X). Then, the cells were separately treated with both classical and El Tor biotypes of *V*. *cholerae* at MOI 10 and incubated overnight at 37°C in an atmosphere containing 5% CO2. Next, the culture medium was removed from each well, and the cells were washed three times with sterile PBS (1X) to remove unattached bacteria from each well. Consequently, the cells and attached bacteria from each well were harvested using RLT solution and transferred into a sterile microtube for RNA extraction [14]. Finally, RNA extraction was performed from treated and untreated (as negative control) Caco-2 cells. For RNA extraction of *V*. *cholerae* in BHI broth culture medium after calculating the attachment percentage based on the bacteria attached to the cells, an equivalent number of bacteria attached to Caco-2 cells was inoculated into the BHI broth medium and incubated overnight at 37°C. After 24 h, RNA was extracted from BHI broth medium separately from both classical and El Tor biotypes of *V*. *cholerae*. Optical absorbance at 260–280 nm was assayed to determine the concentration and purity of the extracted RNA. RNA integrity was investigated using electrophoresis on an agarose gel (1%), which allowed the observation of medium intensity bands without traces of degradation (Figure 1). Finally, cDNA was synthesized using RNA with a concentration of 1.8-2, according to the manufacturer's protocol of Yekta Tajhiz Azma from Iran [15].

### 2.6. Evaluation of the Expression of Virulence Factors of *V*. *cholerae* After Interaction With the Caco-2 Cell Line Using the Real-Time PCR Method

In the current study, the expression of *ctxAB*, *tcpA*, and *hlyA* genes of *V*. *cholerae* was evaluated under two conditions of Caco-2 cell lines and BHI broth environments using the real-time PCR method (ABI System, Applied Biosystems, USA). Briefly, the final reaction volume was 20 μL, containing 1 μL of reverse primer, 1 μL of forward primer, 6 μL of RNase-free water, 2 μL of cDNA, and 10 μL of SYBR Green master mix (Amplicon, Denmark). The primer sequences are detailed in Table 1. The 16s rRNA gene was used as an internal reference in this study. To determine the average ∆CT of each target gene, the CT value of the target genes was subtracted from the average CT of the internal control gene (16s rRNA). Next, the average ∆CT of the control group (classical and El Tor biotypes of *V*. *cholerae* in BHI broth medium) was subtracted from the average ∆CT of target genes to calculate the ∆∆CT value. Subsequently, relative quantification was carried out using standard 2^−ΔΔCT^ analysis. Real-time PCR experiments were carried out in triplicate [15]. The primer sequences are listed in Table 1.

### 2.7. Statistical Analysis

GraphPad Prism version 8 was used for evaluating data using one-way ANOVA and the Bonferroni test. Significance was determined at a *p* value < 0.05. The results are shown as mean ± SD from triplicate experiments.

## 3. Results

### 3.1. Persistence of Caco-2 Cells After Interaction With *V*. *cholerae*

**Figure 2:**
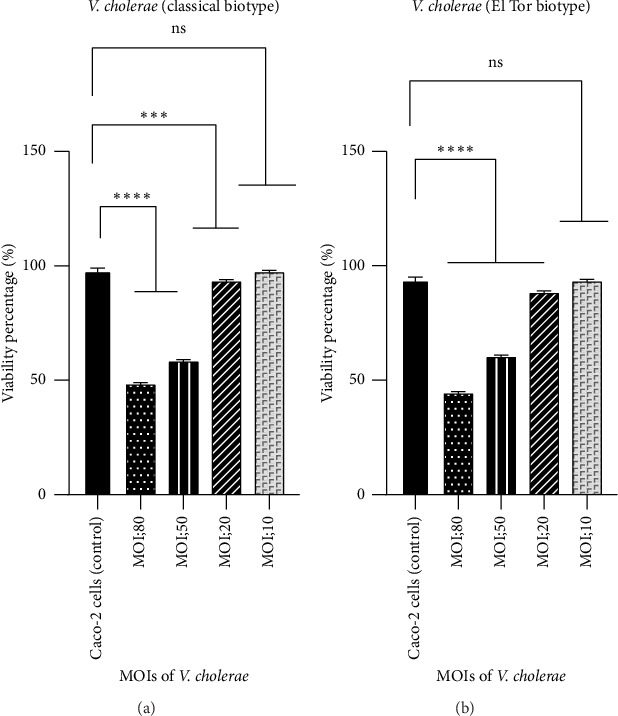
Viability of Caco-2 cells after interaction with *V*. *cholerae*. The classical biotype (a) and the El Tor biotype (b) of *V*. *cholerae* at MOI of 10, 20, 50, and 80. Bars are presented as the mean of triplicate experiments. ^∗∗∗∗^*p* value < 0.0001 and ^∗∗∗^*p* value < 0.0004 were considered statistically significant.

In this study, five groups were investigated including untreated Caco-2 cells (as negative control) and treated cells with *V*. *cholerae* at MOI of 10, 20, 50, and 80. As shown in Figure 2, the viability of Caco-2 cells following interaction with *V*. *cholerae* at MOI of 10, 20, 50, and 80 were 50%, 60%, 91.7%, and 96.6%, respectively. The results indicated that the highest number of live cells was detected after exposure to MOI = 10. So, MOI of 10 as optimal MOI was used.

### 3.2. Evaluation of Attachment of *V*. *cholerae* to Caco-2 Cells

**Figure 3:**
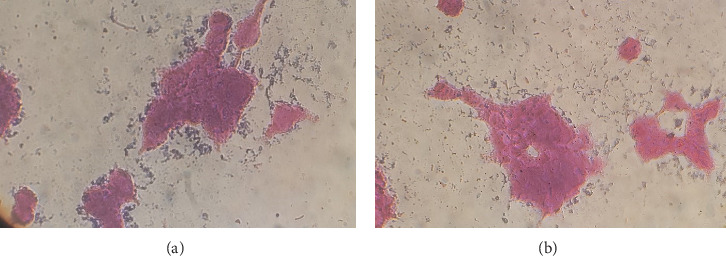
The attachment of classical (a) and El Tor (b) biotypes of *V*. *cholerae* to Caco-2 cells.

Because *V*. *cholerae* does not penetrate inside the cell, we assessed the number of bacteria attached to Caco-2 cells. The equivalent number of *V*. *cholerae* attached to Caco-2 cells was inoculated into BHI broth medium to maintain constant conditions, especially in terms of the bacterial count in both environments. The highest stability of cells was detected at MOI 10, and the attachment test was performed at this MOI. The counts from dilutions (10^−3^ 10^−4^, and 2 × 10^−4^) were 26 × 10^4^ for the classical biotype of *V*. *cholerae* and 22.6 × 10^4^ for the El Tor biotype of *V*. *cholerae*. Therefore, the optimal count from dilution 10^−4^ was then selected for BHI broth inoculation. As shown in Table 2, the percentages of bacteria attached at MOI of 10 in a dilution 10^−4^ to Caco-2 cells in the classical and El Tor biotypes of *V*. *cholerae* were 53% and 44%, respectively (Figures 3(a) and 3(b)).

### 3.3. Examination of the Expression of *ctxAB*, *tcpA*, and *hlyA V*. *cholerae* Genes in Caco-2 Cells and BHI Broth Medium

**Figure 4:**
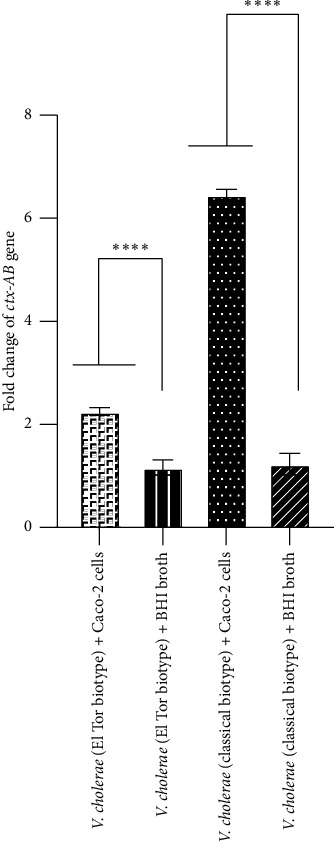
Evaluation of the expression of the *ctxAB* gene using real-time PCR after interaction of Caco-2 cells with El Tor and classical biotypes of *V*. *cholerae* at MOI of 10. Bars are presented as the mean of triplicate experiments. ^∗∗∗∗^*p* value < 0.0001 was considered statistically significant.

**Figure 5:**
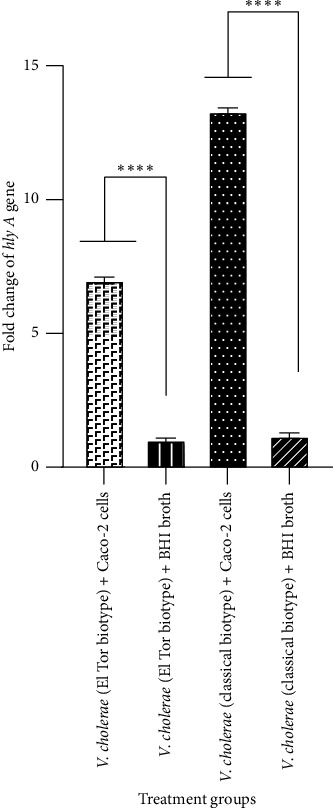
Examination of the expression of the *hlyA* gene using real-time PCR after treatment of Caco-2 cells with El Tor and classical biotypes of *V*. *cholerae* at MOI of 10. Bars are presented as the mean of triplicate experiments. ^∗∗∗∗^*p* value < 0.0001 was considered statistically significant.

**Figure 6:**
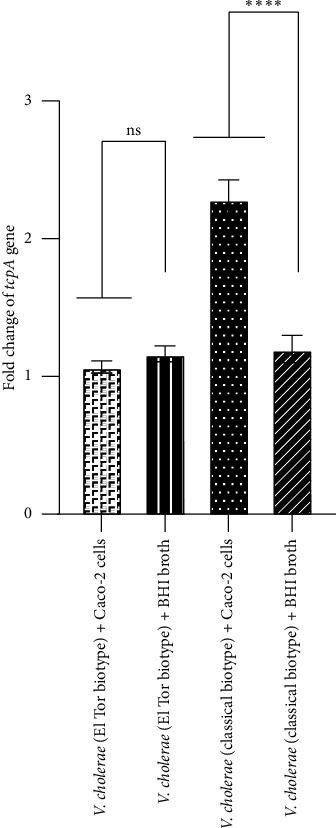
Assessment of the expression of the *tcpA* gene using real-time PCR after exposure of Caco-2 cells with El Tor and classical biotypes of *V*. *cholerae* at MOI of 10. Bars are presented as the mean of triplicate experiments. ^∗∗∗∗^*p* value < 0.0001 was considered statistically significant.

In the current study, the expression of *V*. *cholerae* genes including *ctxAB*, *tcpA*, and *hlyA* after interaction of Caco-2 cells with both El Tor and classical biotypes of *V*. *cholerae* strains was assayed than their expression in BHI broth medium (as a control). Our findings indicated that, after interaction of Caco-2 cells with the El Tor biotype of *V*. *cholerae*, fold changes of *ctxAB*, *tcpA*, and *hlyA* genes were of 2-, 1.02-, and 7-fold, respectively, while fold changes of these genes in the classical biotype of *V*. *cholerae* were 6-, 2-, and 13-fold, respectively. As shown in Figures 4 and 5, there was a significant increase in the expression levels of *ctxAB* and *hlyA* genes in both El Tor and classical biotypes of *V*. *cholerae* after interaction with Caco-2 cells compared to those in BHI broth medium. As shown in Figure 6, after interaction of Caco-2 cells with the classical biotype of *V*. *cholerae*, a significant increase was detected in the expression of the gene *tcpA*, while no significant increase was detected in the expression of this gene in the El Tor biotype. Moreover, after interaction of Caco-2 cells with the classical biotype of *V*. *cholerae*, fold changes of the genes *ctxAB*, *tcpA*, and *hlyA* were higher compared to the El Tor biotype of *V*. *cholerae*. After interaction of Caco-2 cells with the El Tor biotype of *V*. *cholerae*, the expression of the gene *tcpA* was a minimal increase (1.02-fold) than *ctxAB* and *hlyA* genes, while the expression of the gene *hlyA* in the classical biotype *V*. *cholerae* was the highest increase (13-fold) compared to the genes *ctxAB* and *tcpA* (Figure 6). These findings indicated that Caco-2 cell is an enhancer of the expression of the genes *ctxAB*, *tcpA*, and *hlyA* in the classical biotype of *V*. *cholerae*, while it is an enhancer of the genes *ctxAB* and *hlyA* in the El Tor biotype of *V*. *cholerae*. The enhancing effect of Caco-2 cells was more significant on the expression of the genes *ctxAB*, *tcpA*, and *hlyA* in the classical biotype compared to the El Tor biotype of *V*. *cholerae*. However, the enhancing effect of Caco-2 cells on the expression of the genes *ctxAB*, *tcpA*, and *hlyA* in the classical biotype is higher compared to the El Tor biotype of *V*. *cholerae*.

## 4. Discussion

Cholera is a global health challenge, especially in developing nations such as Iran [18]. It is caused by *V*. *cholerae* and leads to severe diarrhea [19]. *V*. *cholerae* inhabits aquatic environments and, under unfavorable conditions, regulates its virulence factors to adapt with the environment [20]. Therefore, a comprehensive understanding from virulence factors and behaviors of *V*. *cholerae* in diverse environmental conditions will facilitate more effective management of this global challenge. Few studies have assessed the effect of Caco-2 cells on the expression of virulence factors in other bacteria. For example, interactions of Caco-2 cells with *Clostridium perfringens* significantly increased the expression of the *Cpe* gene, compared to LB broth medium. They found that Caco-2 cells secrete a substance that suppresses the expression of the *Cpe* gene [7]. Moreover, bacteria have different behaviors in unfavorable conditions, they can impact on the expression of virulence factors, and they can modify the pathogenic ability to adapt to the environment. Key virulence factors of *V*. *cholerae* are CT, toxin-coregulated pilus (*tcpA*), and hemolysin A (*hlyA*) [21]. This study was designed to evaluate the effects of both Caco-2 cells (similar to the intestinal environment) and BHI broth (a suitable laboratory medium) on the expression of the genes *ctxAB*, *tcpA*, and *hlyA* of *V*. *cholerae*. To conduct a valid comparison in this study, after counting the bacteria attached to the Caco-2 cells, this number was inoculated into the BHI broth medium. The main findings of this study following interaction of *V*. *cholerae* with Caco-2 cells include the following: i) there was a significant increase in the expression levels of *ctxAB* and *hlyA* genes in both El Tor and classical biotypes of *V*. *cholerae* compared to those in BHI broth medium; ii) the expression of the gene *tcpA* was increased in the classical biotype of *V*. *cholerae*, while no significant increase was detected in the expression of this gene in the El Tor biotype of *V*. *cholerae*; iii) higher fold changes of *ctxAB*, *tcpA*, and *hlyA* genes were detected in the classical biotype compared to the El Tor biotype of *V*. *cholerae*; iv) the expression of the gene *hlyA* in the classical biotype *of V*. *cholerae* was the highest increase (13-fold) compared to *ctxAB* and *tcpA* genes; and v) no significant increase in the expression of the *tcpA* gene in the El Tor biotype of *V*. *cholerae* was detected (1.02-fold) compared to *ctxAB* and *hlyA* genes.

The results demonstrated that after interaction of the Caco-2 cell with *V*. *cholerae*, fold changes of the genes *ctxAB*, *tcpA*, and *hlyA* in the El Tor biotype of *V*. *cholerae* were lower than fold change in the classical biotype of *V*. *cholerae*. Previous studies indicated that the *hlyA* gene, similar to *ctxAB* and *tcpA* genes of *V*. *cholerae*, is repressed by the quorum sensing (QS) regulated transcription factor including HapR. The process of repression occurs on two distinct levels: transcriptional, independent of HapR, and posttranslational, mediated by HapR [22]. Briefly, the *hly A* gene in the El Tor biotype of *V*. *cholerae* is controlled by the QS system that associated with HapR, FUR, and *hlyA* proteins. HapR binds to the promoters of *hlyA*, hlyU, and FUR for suppressing of the gene *hly A* [22]. On the other hand, *ctxAB* and *tcpA* genes are controlled by the ToxR system, while the El Tor biotype does not express ToxR regulon in the Caco-2 cell environment. Therefore, *ctxAB* and *tcpA* genes are strictly regulated by both HapR and ToxR systems, while *hlyA* is regulated exclusively by the HapR system [23]. It suggested that after interaction of *V*. *cholerae* with Caco-2 cells, the secretory factors produced by the cells led to more suppress of the QS system in *V*. *cholerae*, so after interaction with Caco-2 cells, fold change of the gene *hlyA* in both biotypes is significantly higher than that of the genes *ctxAB* and *tcpA*. However, the fold change of the *hlyA* gene is lower in the El Tor biotype compared to the classical biotype of *V*. *cholerae*. The QS system, mediated by HapR, appears to more strongly repress the expression of the gene *hlyA* in the El Tor biotype compared to the classical biotype of *V*. *cholerae* in the Caco-2 cell environment [23]. Therefore, it is possible that the control of the expression of virulence factors in the El Tor biotype *of V*. *cholerae* be highly complex, potentially involving factors beyond the Tox R regulator. Despite the widely accepted notion that gene expression and protein expression are analogous, a direct correlation between the two is not always evident. In fact, while quantitative assessments of mRNA levels for a specific gene can offer insight into the amount of protein, this correlation is not always observed. A plethora of regulatory mechanisms exist between the process of RNA polymerase traversing the gene and the formation of mRNA and the subsequent protein output from the ribosome. The objective of this study was to determine the expression of *ctxAB*, *tcpA*, and *hlyA* genes in *V*. *cholerae* (El Tor and classical biotypes) after interaction with Caco-2 cells compared to BHI broth. Consequently, it can be deduced that the interpretation of results derived from gene expression analysis should be approached with caution.

However, further research is required in this field, and it is recommended to examine other virulence factors of *V*. *cholerae* in both Caco-2 cells and BHI broth medium.

## 5. Conclusion

In conclusion, this research suggests that changes in environmental conditions can influence the pathogenicity of *V*. *cholerae* which ultimately leads to the organism's adaptation to the environmental conditions. Considering different effects of Caco-2 cells on the expression of *ctxAB*, *tcpA*, and *hlyA* genes in the El Tor and classical biotypes of *V*. *cholerae*, it is possible for the involvement of another regulatory system, potentially beyond HapR and ToxR, in modulating these genes. Therefore, gaining more understanding of how *V*. *cholerae* adapts to adverse conditions by modulating virulence genes can provide novel insight for disease management and prevention strategies.

## Figures and Tables

**Table 1:** The sequence of primers used in real-time PCR.

Gene	Primer-F	Primer-R	References
*ctxAB*	TATGCCAAGAGGACAGAGTGAG	AACATATCCATCATCGTGCCTAAC	[16]
*hlyA*	ACTCGGTTATCGTCAGTTTGG	CGCTTTATTGTTCGATGCGTTA	[17]
*tcpA*	AAGAAAACCGGTCAAGAGGG	TGCGAATCAATCGCACGCTG	This study

**Table 2:** The number of classical and El Tor biotypes of *V*. *cholerae* attachment to Caco-2 cells in three dilutions (10^3^, 10^4^, and 2 × 10^4^).

*V*. *cholerae* strains (MOI:10)	Serial dilution	*V*. *cholerae* attachment to cells (CFU/mL)	Attachment efficiency (%)
Classical biotype (ATCC14035)	10^3^	> 100	—
El Tor biotype	10^3^	> 100	

Classical biotype (ATCC14035)	10^4^	26	53
El Tor biotype	10^4^	22.6	44

Classical biotype (ATCC14035)	2 × 10^4^	12	24
El Tor biotype	2 × 10^4^	9	18

## Data Availability

The data that support the findings of this study are available from the corresponding author upon reasonable request.

## Nomenclature

BHI: Brain heart infusion
*V*. *cholerae*: *Vibrio cholerae*
*ctxAB*: Cholera toxin
*tcpA*: Toxin-coregulated pilus
*hlyA*: Hemolysin A
Zot: Zonula occludens toxin
Ace: Accessory cholera enterotoxin
OmpU: Outer membrane protein U
HapA: Haemagglutinin/protease
ToxR-activated Gene A: TagA cholera toxin lysogenic phage (CTXΦ)
Caco-2: Human colon carcinoma cell line
Cpe: *Clostridium perfringens* enterotoxin
Zn: zinc
Se: Selenium
Mn: Manganese
OD: Optical density
CFU: Colony forming unit
DMEM: Dulbecco's Modified Eagle Medium
FBS: Fetal bovine serum
MOI: Multiplicities of infection
PBS: Phosphate-buffered saline
DMSO: Dimethyl sulfoxide
QS: Quorum sensing.
